# Food insecurity questionnaire on knowledge, attitudes, and practices for perinatal care professionals

**DOI:** 10.1371/journal.pone.0328891

**Published:** 2025-07-21

**Authors:** Gabriela Buccini, Ambree Schoetker, Ana Poblacion, Smriti Neupane, Timothy J. Grigsby, Dodds Simangan, Jyoti Desai, Allison Brown, Juanita Chinn, Manoj Sharma

**Affiliations:** 1 Department of Social and Behavioral Health, University of Nevada, Las Vegas School of Public Health, Paradise, Nevada, United States of America; 2 Children’s HealthWatch, Department of Pediatrics, Boston Medical Center, Boston, Massachusetts, United States of America; 3 Department of Pediatrics, Kirk Kerkorian School of Medicine at UNLV, Las Vegas, Nevada, United States of America; 4 Department of Obstetrics and Gynecology, Kirk Kerkorian School of Medicine at UNLV, Las Vegas, Nevada, United States of America; 5 Division of Cardiovascular Sciences, National Heart Lung and Blood Institutes, National Institutes of Health, Bethesda, Maryland, United States of America; 6 Division of Extramural Research, Population Dynamics Branch, Eunice Kennedy Shriver National Institute of Child Health and Human Development, Bethesda, Maryland, United States of America; 7 Department of Internal Medicine, Kirk Kerkorian School of Medicine at UNLV, Las Vegas, Nevada, United States of America; University of Petra (UOP), JORDAN

## Abstract

**Background:**

Perinatal care professionals are presented with regular opportunities to screen for food insecurity risk and provide referrals for their patients. However, their own experience or lack thereof with food insecurity may interfere with the implementation of screening. Thus, we aimed to develop, validate, and pilot a food insecurity knowledge, attitudes, and practices questionnaire (KAP-FI) for perinatal care professionals that can support the implementation of universal screening for food insecurity.

**Method:**

A multi-step process included (a) questionnaire development, (b) content validation, (c) face validation, (d) reliability and readability, and (e) pilot assessment of KAP-FI. The research team developed the questionnaire; five experts in maternal-child health assisted with content validity, and eight perinatal care professionals assisted with face validity and readability. The pilot phase was carried out with seventy-two perinatal care professionals providing direct services to pregnant people or children under three years in Clark County, Nevada, United States.

**Results:**

KAP-FI included 53 items after content validation, face validation, and pilot phases. Responses from the pilot showed that about 60% of professionals (n = 42) are aware of the 2-item food insecurity screening tool (Hunger Vital Sign™), but of that half (n = 23) indicated that universal screening for food insecurity may not be beneficial to clients/patients. Nonetheless, professionals shared that food insecurity screening (n = 62, 86.1%), referral (n = 69, 95.8%), and follow-up (n = 71, 98.6%) would increase patients trust in them. Thus, 40% (n = 29) reported using a checklist or other reminders to prompt them to screen their clients/patients for food insecurity risk.

**Conclusions:**

KAP-FI is an appropriate and feasible tool to identify baseline barriers and facilitators on knowledge, attitudes, and practices of perinatal care professionals to address food insecurity in the United States.

## Background

Mitigating adversities from pregnancy to early childhood using principles of nurturing care is crucial to promoting human capital and social justice [1]. The Nurturing Care Framework conceptualizes five evidence-based principles (good health, adequate nutrition, safety and security, opportunities for early learning, and responsive caregiving) to provide holistic support for pregnant people and young children [2]. Perinatal interventions that support nurturing care for birthing parents and children can improve health outcomes and support development which in turn is connected to healthcare savings and socioeconomic gains [3]. However, nurturing care can be hindered by stressors such as food insecurity [1,3].

Food insecurity is characterized by the limited or uncertain availability of nutritionally adequate or safe foods or the uncertain ability to acquire acceptable foods in socially acceptable ways [4,5]. Food insecurity during pregnancy has been associated with obesity and gestational diabetes, [6] depression and other psychosocial conditions [7], and low birth weight and prematurity [8]. Experiencing food insecurity during childhood is associated with delays and deficits in development [9–12]. Food insecurity is also linked with increased stress and anxiety for the caregiver, which can hinder their ability to provide nurturing care and respond appropriately to the needs of their child [13,14]. When caregivers are regularly unresponsive to their children’s needs, this can negatively impact early childhood development [15].

Perinatal care professionals including pediatricians, midwives, lactation consultants, doulas, obstetricians and gynecologists, community health workers, and early childhood educators have regular opportunities to screen for food insecurity risk and provide referrals, improve nutrition, and address social needs that directly or indirectly influence disparities in pregnancy and early life [16,17]. Recent efforts to implement strategies to reduce food insecurity in pediatric settings are detailed in the evidence-based toolkit developed by the American Academy of Pediatrics (AAP). The AAP recommends the validated 2-item screening tool Hunger Vital Sign™ to identify families with children at risk for food insecurity [18]. Similar recommendations have been made by the American Academy of Family Physicians [19] the Centers for Medicaid and Medicare Services [20]. However, despite established evidence-based recommendations, many perinatal care professionals do not screen for food insecurity risk [21,22].

Previous studies documented factors limiting the adoption of evidence-based recommendations to address food insecurity, such as lack of awareness of the health consequences of food insecurity, lack of resources to address it, lack of training to screen and refer, time constraints, and challenges with insurance reimbursement [[23–25]. In this context, the knowledge, attitudes, and practices (KAP) framework has been critical to promote the uptake of evidence-based interventions [26,27]. According to the KAP framework, professionals’ behavioral change is achieved through the acquisition of the knowledge, the generation of a way of thinking or feeling (or attitudes), and the adoption of behaviors (or practices) in these three successive processes (i.e., knowledge, attitudes, and practices) [28]. Nonetheless, to date, the KAP of perinatal care professionals to address food insecurity has not been documented. To support the implementation of food insecurity screening processes, this study aimed to develop, validate, and pilot a questionnaire on perinatal care professionals KAP on food insecurity (KAP-FI).

## Methods

### Study design

This exploratory study used a needs assessment design to develop a questionnaire to assess the KAP-FI of perinatal care professionals to inform the implementation of a system-level project to integrate food security interventions for pregnant people and families with young children in five zip codes of Clark County, Nevada, United States. In the associated zip codes of these areas, the population of about 260,044 is 27.5% White, 15.6% Black or African-American, 34.6% Hispanic or Latino, 4.3% two or more races, and 18.0% another race [29].The following steps were involved in establishing the validity of the KAP-FI: (a) questionnaire development, (b) content validation, (c) face validation, (d) reliability and readability, and (e) pilot assessment of KAP-FI with the target population to understand the problems that they face in completing the questionnaire and to receive their suggestions. Ideas for improvements that emerge from this process could be incorporated into further improvement of the questionnaire [30].

### Questionnaire development: item generation and presentation

Based on the purpose of the study, KAP-FI was developed with six sections. The first three (identification and screening, socio-demographics, experience, and workplace) captured professionals’ demographic information, workplace role, and place of work. The remaining three sections assessed knowledge, attitudes, and practices of perinatal care professionals based on the American Academy of Pediatrics (AAP) toolkit for food insecurity screening [18]. Additional items included nutrition-related issues such as concerns about weight or child behavior, and usage of formula due to the United States formula shortage crisis and its relevance to infant food security [31].

The knowledge section captured the breadth of understanding the importance of screening and providing referrals for food insecurity in pregnancy and early childhood, with true or false responses. The attitudes section captured beliefs about screening and referral and its potential impacts on patients or clients, with responses on a four-point Likert scale of agreement: disagree, somewhat disagree, somewhat agree, and agree. Finally, the practices section was intended to capture current behaviors related to food insecurity screening, referral, and follow-up, with responses framed to capture not only current behavior but also plans for future behavior: NO, and I do not intend to in the next 6 months; NO, but I intend to in the next 6 months; NO, but I intend to in the next 30 days; YES, I have been, but for LESS than 6 months; YES, I have been for MORE than 6 months [32]. The initial version of the KAP-FI questionnaire can be found in S1 Table.

### Content validation

The content validation for the questionnaire was based on established methods for KAP questionnaires [30,33–38] and other similar instruments with systematic content validation processes [39–41]. The content validation was conducted in two rounds of reviews by an expert panel. In each round, a panel of experts was asked to rate KAP-FI items based on relevance, and provide qualitative feedback. A summary of content validity methodology can be found in S2 Table.

#### Expert panel review.

Five subject matter experts in fields related to maternal-child health and nutrition participated in content validation. The content experts included three project officers and directors from the National Institutes of Health, one of whom is also a Registered Dietitian Nutritionist, and two Las Vegas physicians who are also faculty at Kirk Kerkorian School of Medicine at the University of Nevada, Las Vegas (UNLV).

#### Data collection.

Instructions were sent via email along with a spreadsheet containing the KAP-FI. Content experts rated item relevance on a 4-point scale: Not relevant [1], Unable to assess relevance without revision [2], Relevant but need minor alterations [3], Very relevant and succinct [4], as well as provided written comments when appropriate. The completed spreadsheets were returned to the research team via email, which were then reviewed item by item.

#### Data analysis.

To obtain content validity index our study estimated the Item-Level and Scale-Level index. For round 1, the Item-Level content validity index (I-CVI) was calculated based on the rating given by each expert for the relevancy of individual items, according to the equation below [42]:


$$I-CVI=\frac{Number\;of\;participants\;scoring\;item\;as\;"Very\;relevant\;and\;succinct"}{Total\;number\;of\;participants}$$

An item was “retained” when the I-CVI was > 0.79, “needed revision” when I-CVI was between 0.70 and 0.79, and “deleted” when I-CVI was < 0.69 [41]. In addition to the quantitative approach, for the items classified as “retained” and “needed revisions”, two research team members (APS, GB) reviewed and addressed qualitative feedback. These feedbacks were thematically classified into structure (confused sentence structure, leading statement, terms not clarified), or technical (item was difficult to understand, overlapping concepts, or too many questions) [38]. For structure feedback, improved wording, enhanced clarity, merged or split, and the items were retained in the questionnaire. For technical feedback, the items were deleted from the questionnaire [38]. Lastly, in round one, new items were added based on qualitative feedback.

For round 2, the I-CVI was estimated for each item (acceptable value at least 0.78). In addition, the Scale-Level content validity index, considering both full scale questionnaire (S-CVI) and sub-scales (SS-CVI) were estimated through two approaches: first, the conservative universal agreement approach (CVI/UA), where the number of items with I-CVI equal to 1 is divided by the total number of items (acceptable value at least 0.60); second, the less conservative average approach (CVI/Ave), where the sum of I-CVIs is divided by the total number of items [43] (acceptable value at least 0.80) [43]. The kappa statistic on inter-rater agreement to supplement the CVI measures was planned to be conducted as a good practice to supplement content validation methodology [43], but it was not able to be calculated due to lack of variability among raters in our study. Content validation individual expert ratings for round 2 are available in S3 Table.

### Face validation

The face validation in our study followed the approach used by previous validated KAP questionnaires [36,37] and similar systematic processes [44–47]. As recommended, for face validation a group of perinatal providers (“test participants”) with similar characteristics of the target population who would eventually respond to the KAP-FI was selected. The test participants’ understanding and interpretation about the items will determine the accuracy of an assessment tool to measure the targeted construct [47]. Test participants were asked to provide qualitative feedback and quantitative ratings based on item clarity, comprehensiveness, and simplicity, [2] calculation of face validity index for Item-Level (I-FVI), Sub-Scales-Level (SS-FIV), and full Scale-Level (S-FVI) were estimated, and [3] items were revised based on participant qualitative feedback. A summary of content validity methodology can be found in S4 Table.

#### Test participants.

Test participants were six physicians in training (i.e., residents) in pediatrics and obstetrics/gynecology, as well as two professionals in early childhood education and maternal-child health serving the Clark County, Nevada zip codes.

#### Data collection.

Consistent with content validation, spreadsheets were sent by email with instructions on how to rate items according to three criteria: clarity (coherent and intelligible), comprehensiveness (including all or nearly all elements of [topic]), and simplicity (easy to understand), with available responses on a 4-point scale: Very clear and succinct [4], Clear but needs minor alterations [3], Unable to assess clarity without revision [2], and Not clear [1], as well as available space for written comments or feedback. Completed spreadsheets were returned to the research team via email and consolidated into one comprehensive spreadsheet.

#### Data analysis.

The face validity index was estimated following the approach proposed by Yusoff [43]. First, the I-FVI was calculated, consisting of the proportion of rater giving an item a clarity, comprehension, and simplicity rating of 3 or 4 by all raters (acceptable value at least 0.80) as indicated in the following formula:


$$I-FVI=\frac{Sum\;of\;participants\;scoring\;item\;as\;4\;or\;3}{\begin{array}{c} Total\;number\;of\;participants \end{array}}$$

The SS and S-FVI were estimated through two approaches: first, the average method (FVI/Ave) consisting of the average of the I-FVI scores for clarity, comprehension, and simplicity for all items on the KAP-FI (acceptable value at least 0.80); second, the universal agreement method (FVI/UA), consisting of the average of the items with I-FVI equal to 1 for clarity, comprehension, and simplicity (acceptable value at least 0.60) [43].

Two research team members (GB, APS) reviewed and addressed any qualitative feedback using the same thematic criteria defined for content validation [38]. The kappa statistic on inter-rater agreement to supplement the FVI measures was planned to be conducted as a good practice to supplement face validation methodology [43], but it was not able to be calculated due to lack of variability among raters in our study. Face validation individual expert ratings are available in S5 Table.

### Reliability and readability

After content and face validation, the reliability and readability of the KAP-FI were assessed. Statistical analyses were conducted using the Statistical Package for Social Sciences (SPSS) Version 28.

To estimate reliability of the KAP-FI, the Cronbach’s alpha coefficient was estimated to measure the KAP-FI internal consistency, i.e., determine whether a collection of items consistently measures the same characteristic. Cronbach’s alpha quantifies the level of agreement on a standardized 0–1 scale, with higher values indicating higher agreement between items [48]. In our study, the internal consistency of the KAP-FI was considered acceptable when the Cronbach’s alpha was above 0.70 [49,50].

To assess the readability, the Flesch-Kincaid readability statistics were estimated to the readability of a text determined by (i) the average length of sentences (measured by the number of words) and (ii) the average number of syllables per word. The Flesch-Kincaid score can range from 0–30 (very difficult to read, best understood by college graduates) to 90–100 (very easy to read, easily understood by an average 11-year-old student) [41]. In our study, the readability of the KAP-FI was considered acceptable if scores range above 30, considering the target population of perinatal care providers.

### Pilot assessment

The KAP-FI questionnaire received ethical approval from the UNLV Institutional Review Board (protocol 1801320-EXE). Written participant consent was obtained before starting the data collection, participation was anonymous, and the privacy of the information was maintained. A financial compensation ($15 Amazon gift card) was provided to participants who completed the survey as a token of appreciation for their time.

#### Participant eligibility.

Individuals 18 years of age and older providing direct services to pregnant people and/or children three years or younger at a community organization located in or adjacent to the area of the study, as well as perinatal care professionals (e.g., doulas, and mental health providers) who travel to the area of the study to provide services took part in the questionnaire pilot. Promotional materials were displayed in their workplaces (e.g., preschools, pediatric, and OB/GYN clinics).

#### Data collection.

The KAP-FI questionnaire was disseminated via email to eligible individuals between August and September 2022. Protocols were created to address formally and systematically potential fraudulent responses from online questionnaires, including repeat responses. For instance, the survey was monitored daily by a research assistant. Any response flagged as duplicate or respondents with location outside of Clark County, Nevada, United States were contacted for a verification process. Three attempts of contact were made; in case of no response to the contact, the response was removed from the database.

#### Data analysis.

Descriptive analysis of sociodemographic characteristics of participants as well as knowledge, attitudes, and practices were conducted using Statistical Package for Social Sciences (SPSS) Version 28.

Sociodemographic characteristics included age (open question, later categorized in 18–30 years; 31–45 years; 46–65 years), gender identity (Man, Woman, Transgender, Non-binary, Two-Spirit, I use a different term; later categorized in Man, Woman, Non-binary/Third-gender), race (American Indian or Alaska Native, Native Hawaiian or other Pacific Islander, some other race, and mixed race), ethnicity (Hispanic, Latino, or Spanish origin), and years of experience in the field (less than 1 year, 1–2 years, 3–5 years 6 years or more). For statistical purposes, race categories with 5 or fewer responses were aggregated with the “other” category. Prefer not to answer responses in any of the sociodemographic characteristics were coded as missing data. Professions were categorized as health (pediatrician, OB/GYN, RN/LPN, midwife, primary care provider, community health worker), nutrition (RD/LDN, nutrition educator, another worker at WIC), mental health (social worker or counselor, other behavioral health worker), supportive care (doula, lactation consultant, parenting educator), and education (early childhood educator and other early childhood worker).

For the knowledge section, the true or false (T/F) responses were coded as correct or incorrect, respectively. For the attitudes section, responses in the 4-point Likert scale were combined for data analysis: agree (agree, somewhat agree), and disagree (disagree, somewhat disagree). For data analysis purposes, in the knowledge and attitudes section, 5 items phrased as negative statements were changed to affirmative statements, and associated responses were properly coded to reflect this change. For the practices section, responses indicating current behavior and future behavioral intention were combined: NO, but I intend to (NO, but I intend to in the next 6 months, and NO, but I intend to in the next 30 days) and YES, and I have been (YES, I have been, but for LESS than 6 months and YES, I have been, but for MORE than 6 months).

## Results

### Questionnaire development

**Fig 1:**
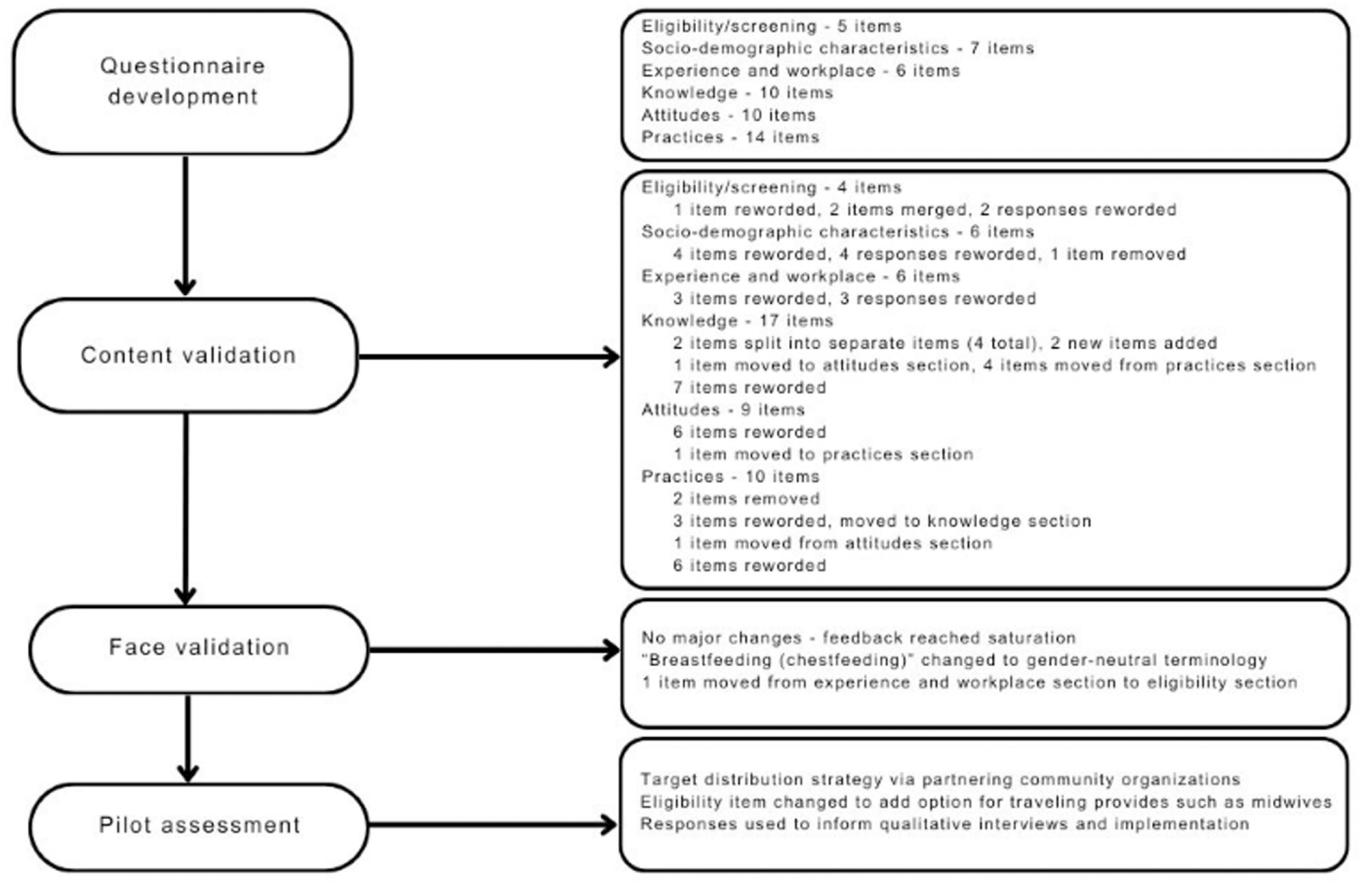
Methods used to develop, validate, and pilot a food insecurity questionnaire on knowledge, attitudes, and practices of perinatal care professionals.

The initial version of KAP-FI consisted of 52 items across six sections: Section 1: identification and screening (5 items), Section 2: socio-demographic characteristics (7 items), Section 3: experience and workplace (6 items), Section 4: knowledge (10 items), Section 5: attitudes (10 items), and Section 6: practices (14 items) (Fig 1). The final version of the KAP-FI questionnaire can be found in S6 Table.

### Content validation

Of the 52 initial items, three were removed based on I-CVI and qualitative feedback (sex assigned at birth and two redundant items in the practices section). Based on qualitative feedback and I-CVI, 28 items were reworded for consistency of language and question format. For example, references to clients or patients were aggregated as “clients/patients” and vague terms such as “food security resources” were specified as either “emergency food resources (e.g., food pantries)” or “food assistance programs (e.g., WIC)”. Two new items were included in the knowledge section due to the United States formula shortage crisis and its relevance to infant food insecurity (“Skilled lactation support provided by food assistance programs (i.e., WIC) may reduce food insecurity among infants” and “Formula feeding of infants among low-income caregivers may increase the risk of infant food insecurity during formula shortages”). Table 1 depicts this process for changes of the questionnaire.

**Table 1:** Content validation changes to the questionnaire on food insecurity knowledge, attitudes, and practices of perinatal care professionals.

Round 1				Round 2		
Item number per section	Item Content	Round 1 I-CVI	Qualitative Feedback	Revised Item Content	Round 2 I-CVI	Qualitative Feedback
**Section 1. Identification and screening**						
Q1	I am 18 years old or older.	1		I am 18 years old or older.	0.8	Structure (minor edits)
Q2	Your PLACE OF WORK or CLINIC zip code: 89030, 89031, 89032, 89101, 89106	1	Structure (minor edits)	Is your work or clinic located within one of these zip codes: 89030, 89031, 89032, 89101, 89106	1	
Q3	Do you provide care or services for pregnant people?	1	Structure (merged)	Do you directly provide care or services for pregnant people and/or children under 3 years old?	1	
Q4	Do you provide care or services for children under 3 years old?	1			1	
Q5	Does the facility where you work accept Medicaid?	1		Does the facility where you work accept Medicaid?	1	
**Section 2. Socio-demographics**						
S1	How old are you?	0.8		How old are you?	1	
N/A	What was the sex assigned to you at birth, on your original birth certificate?	0.8	Structure (minor edits)	What was the sex assigned to you at birth?	0.6	Deleted
S2	What is your current gender?	1		What is your current gender?	1	
S3	What is the highest level of education you have completed?	1		What is the highest level of education you have completed?	1	
S4	Are you Hispanic or Latino/a/x?	1	Structure (minor edits)	Are you of Hispanic, Latino, or Spanish origin?	1	
S5	What race or ethnicity do you consider yourself?	1	Structure (minor edits)	What is your race? Check all that apply.	1	
S6	What language(s) do you speak?	1	Structure (minor edits)	Do you speak a language other than English?	1	
**Section 3. Profession and workplace**						
W1	I provide home visits within the eligible zip codes.	1	Structure (minor edits)	Do you provide home visits?	1	
W2	Within the following zip codes, please indicate the name(s) of the place(s) where you work.	1	Structure (minor edits)	What is the name of the place/clinic you work?	0.8	Structure (minor edits)
W3	What is your employment status at this place?	1		What is your employment status at this place?	1	
W4	How long have you worked at this place?	1		How long have you worked at this place?	1	
W5	I am a (indicate your primary role)	1		What is your primary workplace role?	1	
W6	How long have you been working in your profession?	1		How long have you been working in your profession?	0.8	Structure (minor edits)
**Section 4. Knowledge**						
K1	The Hunger Vital Sign™ is a two-question survey to screen for food insecurity.	0.8	Structure (split into two items)	It is known that households can be screened for food insecurity.	0.8	Structure (minor edits)
K2				I am aware of the Hunger Vital Sign™, a validated food insecurity screening tool that contains two questions.	0.8	Structure (minor edits)
K3	Universal screening for food security is not beneficial to all clients/patients.	1		Screening for food insecurity may not be beneficial to all my clients/patients.	1	
K4	Food assistance programs are the only available resource to address food insecurity within this community.	0.8	Structure (split into two items)	Offices to enroll in food assistance programs (e.g., WIC) are available to my clients/patients within this community.	0.8	Structure (minor edits to make specific to the primary place of work)
K5	Food assistance programs are available to clients living at risk for food security within this community (e.g., SNAP, WIC, CACFP).	1	Structure (minor edits)	For clients/patients already enrolled in food assistance programs (e.g., WIC), they are able to access food within the community thanks to these resources.	0.8	
K6	[added new item to address food resources in the community]	N/A		Organizations providing emergency food resources (e.g., food banks) are available within this community.	0.8	
K7	It is not necessary to refer to WIC when a client/patient is food-insecure and pregnant or caring for a child under 5 years old.	1	Structure (minor edits)	When a client/patient is food-insecure and pregnant or caring for a child under 5 years, I should refer them to enrollment in food assistance programs (e.g., WIC).	1	
K8	Screening positive for food insecurity during pregnancy increases the risk of adverse physical and mental health outcomes (e.g., anxiety, depression, anemia).	1		Screening positive for food insecurity during pregnancy increases the risk of adverse physical and mental health outcomes (e.g., anemia, anxiety, depression).	0.8	Structure (minor edits)
K9	Screening positive and addressing food insecurity during pregnancy decrease the risk of preterm birth and low infant birth weight.	1	Structure (minor edits)	Addressing food insecurity during pregnancy may decrease the risk of preterm birth and low infant birth weight.	1	
K10	[added new item to address breastfeeding resources]	N/A	Structure (new item)	[new] Skilled lactation support provided by food assistance programs (i.e., WIC) may reduce food insecurity among infants.	0.8	Structure (minor edits)
K11	[added new item to address formula shortage]	N/A	Structure (new item)	[new] Formula feeding of infants among low-income caregivers may increase the risk of infant food insecurity during formula shortages.	0.8	Structure (minor edits)
K12	Screening positive and addressing food insecurity during pregnancy and/or after birth is likely to decrease milk production for breastfeeding (chestfeeding.)	0.8	Structure (minor edits)	Food insecurity during pregnancy and/or after birth is likely to decrease milk production for breastfeeding.	0.8	Structure (minor edits)
K13	Screening positive and addressing food insecurity during childhood does not impact child development.	1	Structure (minor edits)	Addressing food insecurity during childhood may not impact child development.	0.8	Structure (minor edits)
K14	Screening for and addressing food insecurity among my clients/patients is likely to positively impact the health and wellbeing of my patients and their families.	1	Structure (split into four items)	It is not important to screen for food insecurity during patient/client visits for nutrition-related conditions (e.g., diabetes, weight concerns, food allergies).	1	
K15				It is not important to screen for food insecurity when a child age 3 or younger has behavioral problems.	1	
K16				It is not important to screen for food insecurity when a patient/client requires a special diet.	1	
K17				It is not important to screen for food insecurity when a patient/client requires expensive medication.	1	
**Section 5. Attitudes**						
A1	I believe that addressing food insecurity is an important part of my profession.	1	Structure (minor edits)	Screening for and addressing food insecurity is likely to positively impact the physical and mental health of my clients/patients and their families. Therefore, I am ready to do my part.	1	
A2	Providing clients/patients with referrals to food security resources is not in my scope of practice.	1	Structure (minor edits)	Providing clients/patients with referrals to emergency food resources (e.g., food banks) and/or food assistance programs (e.g., WIC) to reduce food insecurity is not in my scope of practice.	0.8	Structure (minor edits)
A3	I am comfortable screening my clients/patients for food insecurity.	1		I am comfortable screening my clients/patients for food insecurity.	1	
A4	I am comfortable referring my clients/patients to food security resources.	1	Structure (minor edits)	I am comfortable referring my clients/patients to emergency food resources (e.g., food banks) and/or food assistance programs (e.g., WIC).	0.8	Structure (minor edits)
A5	A universal screening tool for food insecurity is/would be useful in my practice.	1		A universal screening tool for food insecurity is/would be useful in my practice.	1	
A6	My workload allows for sufficient time to screen clients/patients for food insecurity.	1	Structure (minor edits)	Universal screening for food insecurity interferes/would interfere with my routine.	1	
A7	My clients/patients would trust me more if I screened them for food insecurity.	1		My clients/patients would trust me more if I provided them with referrals to community resources to reduce food insecurity.	1	
A8	My clients/patients would trust me more if I provided them with referrals to food security resources.	1		My clients/patients would trust me more if I screened them for food insecurity.	1	
A9	My clients/patients would trust me more if I followed up with them after referral to food security resources.	1		My clients/patients would trust me more if I followed up with them after referral to community resources to reduce food insecurity.	1	
**Section 6. Practices**						
P1	I screen all pregnant or postpartum clients/patients for food insecurity.	0.8		I screen all pregnant or postpartum clients/patients for food insecurity.	0.8	Structure (minor edits)
P2	The electronic medical record system that I use is conducive to universal food insecurity screening. [Item moved from Section 5. Attitudes]	1	Structure (minor edits)	I use an electronic record system to screen for food insecurity.	1	
P3	I screen clients/patients (or their caregiver for children up to the age of 3) for food insecurity during all interactions.	1		I screen clients/patients (or their caregiver for children up to the age of 3 years) for food insecurity during all interactions.	0.8	Structure (minor edits)
P4	I coordinate with community resources to reduce food insecurity among my clients/patients.	1		I coordinate with community resources to reduce food insecurity among my clients/patients.	1	
P5	I refer families experiencing food insecurity to food security services.	1	Structure (minor edits)	I refer families experiencing food insecurity to food assistance programs (e.g., WIC) and/or emergency food resources (e.g., food banks).	1	
P6	After referring a client/patient or family to food security services, I follow up to ensure that food needs are met.	1	Structure (minor edits)	After referring a client/patient or family to food assistance programs (e.g., WIC) and/or emergency food resources (e.g., food bank), I follow up to ensure that food needs are met.	1	
P7	When I feel like I do not have time to screen for food insecurity, I make myself anyway because I know it will make a difference to me and my client/patients.	1		When I feel like I do not have time to screen for food insecurity, I make myself anyway because I know it will make a difference to my clients/patients.	1	
P8	I schedule meetings and events to educate my clients/patients about food security resources in the community.	1	Structure (minor edits)	I schedule meetings and events to educate my clients/patients in the community about food assistance programs (e.g., WIC) and/or emergency food resources (e.g., food banks) to reduce food insecurity.	1	
P9	I have posters and educational materials about food security visible in areas frequented by clients/patients.	1		I have posters and educational materials about food insecurity visible in areas frequented by clients/patients.	1	
P10	I use a checklist or other form of reminder to prompt me to screen my clients/patients for food insecurity.	1		I use a checklist or other form of reminder to prompt me to screen my clients/patients for food insecurity.	1	
N/A	I screen for food insecurity when a child age 3 or younger has behavioral problems.	0.7	Technical (excluded due to overlapping questions and deviating from universal screening)			
N/A	I screen for food insecurity when a patient/client requires a special diet or expensive medication.	0.7				
N/A	I screen for food insecurity during visits for nutrition-related conditions (e.g., diabetes, weight concerns, food allergies).	0.7				
N/A	I screen for food insecurity in patients with depression and/or anxiety.	0.7				
N/A	I screen for food insecurity in patients with anemia.	0.7				

At the end of the content validation phase, the KAP-FI questionnaire contained 53 items. According to the S-CVI/Ave-, the questionnaire scored 0.93; whereas, according to the S-CVI/UA, the questionnaire scored 0.69 (Table 2).

**Table 2:** Content and face validation methodology for the questionnaire on food insecurity knowledge, attitudes, and practices of perinatal care professionals.

KAP-FI questionnaire	Content Validity Index Average (CVI/Ave)	Content Validity Universal Agreement (CVI/UA)	Face Validity Index Average (FVI/Ave)	Face Validity Universal Agreement (FVI/UA)
Full scale (n = 53 items)	0.93	0.69	0.99	0.64
Sub-scale: Knowledge (n = 17 items)	0.88	0.41	0.99	0.94
Sub-scale: Attitudes (n = 9 items)	0.96	0.78	0.99	0.88
Sub-scale: Practices (n = 10 items)	0.96	0.80	0.97	N/A*

* Not Available due to lack of universal agreement.

### Face validation

When all items scored above 0.875 in I-CVI, feedback reached saturation. This corresponded to endorsement of at least 7 of 8 raters. According to the S-FVI/Ave, the questionnaire scored 0.99; whereas according to the S-FVI/UA, the questionnaire scored 0.64 (Table 2).

Table 3 outlines the face validation and process for changes to the questionnaire. A small change unrelated to content was implemented to avoid “othering” transmasculine individuals and for consistency with the Academy of Breastfeeding Medicine’s recommendation of using inclusive language in regard to gender identity and lactation [47] (“breastfeeding (chestfeeding)” was aggregated as “breast/chestfeeding”).

**Table 3:** Face validation methodology for the questionnaire on food insecurity knowledge, attitudes, and practices of perinatal care professionals.

Item number	Section & Item	I-FVI Clarity	I-FVI Simplicity	I-FVI Comprehensiveness	Qualitative Feedback
**Section 1. Identification and screening**					
** Q1**	Are you 18 years old or older?	1.00	1.00	1.00	
** Q2**	Is your primary workplace or clinic located within one of these zip codes: 89030, 89031, 89032, 89101, 89106	0.88	0.88	0.88	Structural (removed the word “clinic”)
** Q3**	Do you directly provide care or services for pregnant people and/or children under 3 years old?	0.75	1.00	0.88	Structural (added, e.g.,)
** Q4**	Does the facility where you work accept Medicaid?	1.00	1.00	1.00	
**Section 2. Socio-demographics**					
** **S1	How old are you?	1.00	1.00	1.00	
** S2**	What is your current gender?	1.00	1.00	0.88	
** S3**	What is the highest level of education you have completed?	1.00	1.00	1.00	
** S4**	Are you of Hispanic, Latino, or Spanish origin?	1.00	1.00	1.00	
** S5**	What is your race? Check all that apply.	1.00	1.00	1.00	
** S6**	Do you speak a language other than English?	1.00	1.00	0.88	
**Section 3. Profession and workplace**					
** W1**	Do you provide home visits?	0.88	0.88	0.88	Structural (clarified)
** W2**	What is the name of your primary workplace?	1.00	0.88	0.88	
** W3**	What is your employment status at this place?	1.00	1.00	1.00	
** W4**	How long have you worked at this place?	1.00	1.00	1.00	
** W5**	What is your primary workplace role?	0.88	0.75	0.88	Structural (wording)
** W6**	How long have you been working in this profession?	1.00	1.00	1.00	
**Section 4. Knowledge**					
** K1**	It is possible to screen households for food insecurity.	1.00	1.00	1.00	
** K2**	I am aware of the Hunger Vital Sign™, a validated food insecurity screening tool that consists of two questions.	1.00	1.00	1.00	
** K3**	Screening for food insecurity may not be beneficial to all my clients/patients.	1.00	1.00	1.00	
** K4**	Offices to enroll in food assistance programs (e.g., WIC) are available to my clients/patients within this community.	1.00	1.00	1.00	
** K5**	For clients/patients already enrolled in food assistance programs (e.g., WIC), they are able to access food within the community thanks to these resources.	1.00	1.00	1.00	
** K6**	Organizations providing emergency food resources (e.g., food banks) are available within this community.	1.00	1.00	1.00	
** K7**	When a client/patient is food-insecure and pregnant or caring for a child under 5 years, I should refer them to enrollment in food assistance programs (e.g., WIC).	1.00	1.00	1.00	
** K8**	Experiencing food insecurity during pregnancy increases the risk of adverse physical and mental health outcomes (e.g., anemia, anxiety, depression).	1.00	1.00	1.00	
** K9**	Addressing food insecurity during pregnancy may decrease the risk of preterm birth and low infant birth weight.	1.00	1.00	1.00	
** K10**	Breastfeeding (chestfeeding) support provided by food assistance programs (i.e., WIC) may reduce food insecurity among infants.	1.00	1.00	1.00	
** K11**	Formula feeding instead of breastfeeding (chestfeeding) among low-income families may increase risk of infant food insecurity, especially during formula shortages.	1.00	0.88	1.00	Structural (removed word)
** K12**	Addressing food insecurity during childhood will not have a positive impact on child development.	1.00	1.00	1.00	
** K13**	Experiencing food insecurity during pregnancy and/or after birth is likely to decrease milk production for breastfeeding (chestfeeding.)	1.00	1.00	1.00	
** K14**	It is not important to screen for food insecurity during patient/client visits for nutrition-related conditions (e.g., diabetes, weight concerns, food allergies).	1.00	1.00	1.00	
** K15**	It is not important to screen for food insecurity when a child age 3 or younger has behavioral problems.	1.00	1.00	1.00	
** K16**	It is not important to screen for food insecurity when a patient/client requires a special diet.	1.00	1.00	1.00	
** K17**	It is not important to screen for food insecurity when a patient/client requires expensive medication.	1.00	1.00	1.00	
**Section 5. Attitudes**					
** A1**	Screening for and addressing food insecurity is likely to positively impact the physical and mental health of my clients/patients and their families. Therefore, I am ready to do my part.	1.00	1.00	0.88	
** A2**	Providing clients/patients with referrals to food assistance programs (e.g., WIC) and/or emergency food resources (e.g., food banks) is not in my scope of practice.	1.00	1.00	1.00	
** A3**	I am comfortable screening my clients/patients for food insecurity.	1.00	1.00	1.00	
** A4**	I am comfortable referring my clients/patients to food assistance programs (e.g., WIC) and/or emergency food resources (e.g., food banks).	1.00	1.00	1.00	
** A5**	A universal screening tool for food insecurity is/would be useful in my practice.	1.00	1.00	1.00	
** A6**	Universal screening for food insecurity interferes/would interfere with my routine.	1.00	1.00	1.00	
** A7**	My clients/patients would trust me more if I screened them for food insecurity.	1.00	1.00	1.00	
** A8**	My clients/patients would trust me more if I provided them with referrals to community resources to reduce food insecurity.	1.00	1.00	1.00	
** A9**	My clients/patients would trust me more if I followed up with them after referral to community resources to reduce food insecurity.	1.00	1.00	1.00	
**Section D. Practices**					
** P1**	I screen all pregnant or postpartum clients/patients for food insecurity.	0.88	0.88	0.88	
** P2**	I screen clients/patients or their caregiver for children up to the age of 3 years for food insecurity during all interactions.	0.88	0.88	0.88	
** P3**	I use an electronic record system to screen for food insecurity.	0.88	0.88	0.88	
** P4**	I coordinate with community resources to reduce food insecurity among my clients/patients.	0.88	0.88	0.88	
** P5**	I refer families experiencing food insecurity to food assistance programs (e.g., WIC) and/or emergency food resources (e.g., food banks).	0.88	0.88	0.88	
** P6**	After referring a client/patient or family to food assistance programs (e.g., WIC) and/or emergency food resources (e.g., food banks), I follow up to ensure that food needs are met.	0.88	0.88	0.88	
** P7**	When I feel like I do not have time to screen for food insecurity, I make myself anyway because I know it will make a difference to my clients/patients.	0.88	0.88	0.88	
** P8**	I schedule meetings and events to educate my clients/patients in the community about food assistance programs (e.g., WIC) and/or emergency food resources (e.g., food banks) to reduce food insecurity.	0.88	0.88	0.88	
** P9**	I have posters and educational materials about food insecurity visible in areas frequented by clients/patients.	0.88	0.88	0.88	
** P10**	I use a checklist or other form of reminder to prompt me to screen my clients/patients for food insecurity.	0.88	0.88	0.88	
**S-FVI/Ave**		**0.95**	**0.94**	**0.94**	
**Total agreement**		**38**	**37.00**	**34.00**	
**S-FVI/UA**		**0.72**	**0.70**	**0.64**	

### Reliability and readability

Internal consistency of KAP-FI exhibited Cronbach’s alpha coefficient of 0.85. The Flesch-Kincaid Reading Ease Score was 43, requiring a grade level of 11.3 years of education to read and understand the questionnaire.

### Pilot assessment

Seventy-two individuals completed the survey. Most participants identified as women (n = 61, 68.1%), half were between 31 and 45 years old (n = 36, 50.0%), about one-third were Spanish speakers (n = 27, 37.5%), and approximately 40% (n = 29) had more than six years of work experience. Participants were racially diverse with 37.5% White, 23.6% Asian, and 19.5% Black/African American (Table 4). Based on self-reported profession and the Nurturing Care Framework, about half were in the domain of good health (n = 35, 48.6%) (Table 4).

**Table 4:** Socio-demographic characteristics of perinatal care professionals who participated in the pilot assessment of the questionnaire on food insecurity knowledge, attitudes, and practices.

Sociodemographic characteristics	N (%)
**Race**	
White	27 (37.5)
Black or African American	14 (19.4)
Asian	17 (23.6)
Other	9 (12.5)
Prefer not to answer	5 (6.9)
**Ethnicity**	
Hispanic/Latino	22 (30.6)
Non-Hispanic/Latino	49 (68.1)
Prefer not to answer	1 (1.4)
**Gender**	
Man	9 (12.5)
Woman	61 (84.7)
Non-binary/Third gender	2 (2.8)
**Age**	
18-30 years	22 (30.6)
31-45 years	36 (50.0)
46-65 years	14 (19.4)
**Years of experience in the profession**	
Less than 1 year	9 (12.5)
1-2 years	15 (20.8)
3-5 years	19 (26.4)
6 years or more	29 (40.3)
**Professions according to the Nurturing Care Framework**	
Good Health	35 (48.6)
*Pediatrician*	*9 (12.5)*
*OB/GYN*	*8 (11.1)*
*Primary care provider*	*5 (6.9)*
*RN/LPN*	*5 (6.9)*
*Community health worker*	*5 (6.9)*
*Midwife*	*3 (4.2)*
Safety and Security	11 (15.3)
*Mental health professional*	*7 (9.7)*
*Other behavioral health worker*	*4 (5.6)*
Adequate nutrition	10 (13.9)
*Nutrition educator*	*5 (6.9)*
*Another worker at WIC*	*3 (4.2)*
*RD/LDN*	*2 (2.8)*
Responsive caregiving	9 (12.5)
*Doula*	*4 (5.6)*
*Lactation consultant*	*3 (4.2)*
*Parenting educator*	*2 (2.8)*
Opportunities for early learning	7 (9.7)
*Early childhood educator*	*5 (6.9)*
*Other early childhood worker*	*2 (2.8)*

In the knowledge section (Table 5), more than half of the participants (n = 42, 58.3%) reported awareness of the 2-item food insecurity screen Hunger Vital Sign™; however, nearly a third (n = 23, 31.9%) indicated that screening for food insecurity may not be beneficial to all clients/patients, and nearly one quarter (n = 16, 22.2%) reported that food insecurity screening is not important during visits for nutrition-related conditions (e.g., diabetes, weight concerns, food allergies).

**Table 5:** Pilot assessment responses: knowledge.

Questionnaire item	Correct N (%)	Incorrect N (%)
It is possible to screen households for food insecurity.	65 (90.3)	7 (9.7)
I am aware of the Hunger Vital Sign™, a validated food insecurity screening tool that consists of two questions.	30 (41.7)	42 (58.3)
Screening for food insecurity may not be beneficial to all my clients/patients.	49 (68.1)	23 (31.9)
Offices to enroll in food assistance programs (e.g., WIC) are available to my clients/patients within this community.	69 (95.8)	3 (4.2)
For clients/patients already enrolled in food assistance programs (e.g., WIC), they are able to access food within the community thanks to these resources.	67 (93.1)	5 (6.9)
Organizations providing emergency food resources (e.g., food pantries) are available within this community.	68 (94.4)	4 (5.6)
When a client/patient is food-insecure and pregnant or caring for a child under 5 years, I should refer them to enrollment in food assistance programs (e.g., WIC).	71 (98.6)	1 (1.4)
Experiencing food insecurity during pregnancy increases the risk of adverse physical and mental health outcomes (e.g., anemia, anxiety, depression).	71 (98.6)	1 (1.4)
Addressing food insecurity during pregnancy may decrease the risk of preterm birth and low infant birth weight.	71 (98.6)	1 (1.4)
Lactation support provided by food assistance programs (i.e., WIC) may reduce food insecurity among infants.	72 (100)	0 (0)
Formula feeding instead of breast/chestfeeding among low-income families may increase risk of food insecurity, especially during formula shortages.	65 (90.3)	7 (9.7)
Experiencing food insecurity during pregnancy and/or after birth is likely to decrease milk production for breast/chest feeding.	9 (12.5)	63 (87.5)
Addressing food insecurity during childhood will not have a positive impact on child development.	54 (75.0)	18 (25.0)
It is important to screen for food insecurity during patient/client visits for nutrition-related conditions (e.g., diabetes, weight concerns, food allergies).^*^	56 (77.8)	16 (22.2)
It is important to screen for food insecurity when a child age 3 or younger has behavioral problems.^*^	63 (87.5)	9 (12.5)
It is important to screen for food insecurity when a patient/client requires a special diet.^*^	65 (90.3)	7 (9.7)
It is important to screen for food insecurity when a patient/client requires expensive medication.^*^	68 (94.4)	4 (5.6)

* Note: In the pilot version of KAP-FI, these items were written with a negative frame, later changed to reflect affirmative statements.

In the attitudes section (Table 6), most participants (n = 59, 81.9%) reported that universal screening for food insecurity would not interfere with their routine. Participants indicated that their involvement in food insecurity screening (n = 62, 81.1%), referral (n = 69, 95.8%), and follow-up (n = 71, 98.6%) would increase trust in the relationship with clients/patients.

**Table 6:** Pilot assessment responses: attitudes.

Questionnaire item	Agree N (%)	Disagree N (%)
Screening for and addressing food insecurity is likely to positively impact the physical and mental health of my clients/patients and their families. Therefore, I am ready to do my part.	71 (98.6)	1 (1.4)
Providing clients/patients with referrals to food assistance programs (e.g., WIC) and/or emergency food resources (e.g., food pantries) is in my scope of practice.^*^	62 (86.1)	10 (13.9)
I am comfortable screening my clients/patients for food insecurity.	63 (87.5)	9 (12.5)
I am comfortable referring my clients/patients to food assistance programs (e.g., WIC) and/or emergency food resources (e.g., food pantries).	67 (93.1)	5 (6.9)
A universal screening tool for food insecurity is/would be useful in my practice.	70 (97.2)	2 (2.8)
Universal screening for food insecurity interferes/would interfere with my routine.	13 (18.1)	59 (81.9)
My clients/patients would trust me more if I screened them for food insecurity.	62 (86.1)	10 (13.9)
My clients/patients would trust me more if I provided them with referrals to community resources to reduce food insecurity.	69 (95.8)	3 (4.2)
My clients/patients would trust me more if I followed up with them after referral to community resources to reduce food insecurity.	71 (98.6)	1 (1.4)

* In the pilot version of KAP-FI, this item was written with a negative frame, later changed to reflect an affirmative statement.

In the practices section (Table 7), almost half (n = 32, 44.4%) reported currently screening all pregnant or postpartum clients/patients for food insecurity risk. Slightly more than half (n = 38, 52.8%) indicated screening households with children younger than three years for food insecurity risk in every visit. Less than 30% (n = 20, 27.8%) described using an electronic record system to screen for food insecurity risk. About 40% (n = 29) reported using a checklist or other form of reminder to prompt them to screen their clients/patients for food insecurity risk. About one-third (n = 24, 33.3%) shared that their workplace had posters or educational materials about food insecurity visible to clients/patients.

**Table 7:** Pilot assessment responses: practices.

Questionnaire item	NO, and I do not intend to N (%)	NO, but I intend to N (%)	YES, and I have been N (%)
I screen all pregnant or postpartum clients/patients for food insecurity.	9 (12.5)	31 (43.1)	32 (44.4)
I screen clients/patients or their caregivers for children up to the age of 3 years for food insecurity during all interactions.	5 (6.9)	29 (40.3)	38 (52.8)
I use an electronic record system to screen for food insecurity.	17(23.6)	35 (48.6)	20 (27.8)
I coordinate with community resources to reduce food insecurity among my clients/patients.	10 (13.9)	24 (33.3)	38 (52.8)
I refer families experiencing food insecurity to food assistance programs (e.g., WIC) and/or emergency food resources (e.g., food pantries).	3 (4.2)	9 (12.5)	60 (83.3)
After referring a client/patient or family to food assistance programs (e.g., WIC) and/or emergency food resources (e.g., food pantries), I follow up to ensure that food needs are met.	7 (9.7)	27 (37.5)	38 (52.8)
When I feel like I do not have time to screen for food insecurity, I make myself anyway because I know it will make a difference to my clients/patients.	8 (11.1)	24 (33.3)	40 (55.6)
I schedule meetings and events to educate my clients/patients in the community about food assistance programs (e.g., WIC) and/or emergency food resources (e.g., food pantries) to reduce food insecurity.	22 (30.6)	24 (33.3)	26 (36.1)
I have posters and educational materials about food insecurity visible in areas frequented by clients/patients	17 (23.6)	31 (43.1)	24 (33.3)
I use a checklist or other form of reminder to prompt me to screen my clients/patients for food insecurity.	13 (18.1)	30 (41.7)	29 (40.3)

## Discussion

To our knowledge, KAP-FI represents the first attempt to systematize a quantitative assessment of a KAP questionnaire for perinatal care professionals when addressing food insecurity risk among their clients/patients during pregnancy and early childhood. To do so, a systematic and pragmatic process aimed at creating a reliable, comprehensive, and easy-to-use questionnaire was developed. The pilot assessment demonstrated KAP-FI’s reliability and appropriateness in assessing barriers and facilitators within three sub-scales (i.e., knowledge, attitudes, and practices) of professional readiness to implement evidence-based recommendations to address food insecurity. KAP-FI may be a useful tool to inform strategies to close the current gap of implementation by increasing the uptake of evidence-based recommendations to screen and intervene for food insecurity during pregnancy and early childhood in the United States.

The development of KAP-FI followed best practices for designing this type of survey, including (i) the development of questions, answers, and scoring, and (ii) the validation process [30]. A careful process for developing the questions, answers, and scoring systems involved a review of existing literature on evidence-based guidelines for addressing food insecurity in perinatal care settings in the United States as well as a participatory and consultative process that combined qualitative and quantitative assessments with a diverse group of stakeholders. As a result, the final KAP-FI had a reliable internal consistency among the set of survey items, and the readability level was found compatible with the target population, which are skilled perinatal care professionals with at least a high school degree. These validation features strengthen the appropriateness of the KAP-FI questionnaire to assess baseline knowledge, attitudes, and practices of perinatal care professionals on food insecurity among the maternal-child population in the United States.

While developing items, a topical subject addressed in the questionnaire was the potential impact of a shortage of infant formula [31] on food security. Because low-income populations may be more likely to be impacted by formula shortages due to restricted ability to breast/chest feed (e.g., lack of paid parental leave) and limited purchasing power, educating perinatal care professionals on this topic is critical. Viewing food insecurity through the lens of improving maternal-child health compels support and advocacy for exclusive breast/chestfeeding, including support of policies and socioeconomic conditions that uplift and empower families to make healthy choices with the guidance of qualified professionals [17].

KAP-FI validation involved content and face validation followed by an additional step of piloting testing in the target population, which are recommended steps for designing KAP surveys in prior studies [30,51]. Following the best practices to assess questionnaire validity, we calculated both content and face validation indexes to capture validity of Item-Level, Sub-Scales-Level (i.e., knowledge, attitudes, and practices), and Scale-Level [30,51]. However, construct and criterion validity were not assessed in this study; thus, opportunities to strengthen the KAP-FI validation including psychometric tests such as Rasch analysis and construct validation using confirmatory factor analysis exist and may be pursued in a future study. For the present study, both validation approaches were adapted to be pragmatic for community-based research and allowed for the incorporation of suggestions and ideas from local clinicians and leaders of community organizations who are experienced in their relative areas to improve the questionnaire. In turn, that enhanced the reliability and appropriateness of the questionnaire in informing food insecurity screening implementation plans using facilitators as opportunities to address barriers, which were the major initial goals to develop KAP-FI.

Indeed, this pilot assessment demonstrated the reliability and appropriateness of KAP-FI to support the identification of barriers and facilitators to inform food insecurity screening implementation in perinatal care settings. It was possible to identify knowledge barriers that may hinder screening implementation including lack of awareness of the Hunger Vital Sign™ and lack of understanding that screening can benefit all clients/patients. Identified knowledge facilitators that may support screening implementation include an understanding of adverse health outcomes linked to food insecurity and awareness of validated screening tools. Based on findings, knowledge-based strategies can be developed to increase awareness of health outcomes associated with food insecurity [52,53] and systematize a screening process with a robust referral network to connect individuals to resources that alleviate food insecurity [54].

Attitudes were identified that could present barriers to screening, including lack of comfort with raising the subject of food insecurity and the belief that addressing food insecurity is outside of the professional’s scope of practice. Attitudes that may facilitate implementation of screening included the belief that addressing food insecurity through screening, referrals to community resources, and follow-up after referrals would increase trust. Based on KAP-FI findings supported by prior research, attitudes-based strategies would include training for professionals to reduce the sensitivity or stigma around food insecurity [52], developing systems for follow-up after referrals [52,55,56] and navigating social welfare and safety net programs [57,58].

Practices identified in this study that may create barriers to the implementation of food insecurity screening include not using an electronic record system, challenges navigating paperwork and eligibility criteria for social programs and community resources, and lack of coordination with community partners/resources. Practices that may facilitate implementation of food insecurity screening include the use of a checklist and having posters and educational materials about food insecurity in areas visible to clients/patients. Based on the KAP-FI findings, strategies would include evidence-based practices such as the use of an electronic record system [59,60], navigation and coordination with community and social resources, collaboration and community partnerships to advance health equity in populations disproportionately affected by food insecurity [61], the use of checklists for quality improvement [62], including the use of posters or educational materials about food insecurity visible to clients/patients [18].

Although KAP-FI allows for collection of valuable information, there are potential limitations to be considered when interpreting results as well as in its future application. The validation process relied on similar Item-Level, Sub-Scale-Level, and Scale-Level content and face validity indexes. Although different raters were used for content and face validation, three participants from each group rated the questionnaire with perfect scores (no revisions recommended), and thus the kappa statistic could not be calculated due to high inter-rater agreement. These limitations are addressed through other calculations, including Sub-Scale- Level and Scale-Level validity indexes using both the average and universal agreement methods, which are less conservative methods than the kappa statistic.

The KAP model has been widely used due to being an easy and cost-effective way of assessing important dimensions for implementation. However, there are at least two important considerations. First, there is the question of generalizability. In this pilot assessment, the sample of professionals was limited to those providing nurturing care services in the areas of Clark County, Nevada, a racially and ethnically diverse urban community. Therefore, other populations may respond differently; therefore, future validation in larger samples is needed to confirm generalizability of the KAP-FI. Additionally, this pilot assessment relied primarily on responses from professions working in Nurturing Care frameworks of “good health” and “adequate nutrition”; therefore, future studies should expand to other Nurturing Care areas by increasing participation from professionals who engage in case management with socioeconomically vulnerable families (e.g., social workers in child protective services).

Second, it is assumed that there is a direct relationship between knowledge, attitude, and practices [63,64]. It is imprecise to assume that a sample is representative of the social and cultural attitudes of an entire population and especially that knowledge and attitudes are directly correlated with the decisions and behaviors of a person or how a community functions. The growing literature on population health innovations in screening, referral, and social service integration [65–68] offers new opportunities to address food insecurity using health behavior theories that have been rigorously tested to make broader generalizations. Future work could consider theoretical justification (including construct validation), revising KAP-FI, conducting additional psychometric tests, and piloting KAP-FI in other regions (including retrospective validation, such as through review of health record data, and prospective validation, such as through pre-tests prior to implementation of screening).

In conclusion, KAP-FI was successfully validated to identify barriers and facilitators to food insecurity screening, referral, and subsequent follow-up in nurturing care settings. The KAP model strengthens the development of the questionnaire, making it easily applicable in the daily routines of professionals. The evidence-based validation approach resulted in a reliable and appropriate questionnaire to address and overcome barriers, as well as identify benefit from facilitators to increase the chances of successful implementation of food insecurity screening.

## Supporting information

S1 Table Initial Version of the Questionnaire on Food Insecurity Knowledge, Attitudes, and Practices for Perinatal Care Professionals, Prior to Round 1 of Content Validation. (DOCX)

S2 Table Content Validity Methodology for the Food Insecurity Questionnaire on Knowledge, Attitudes, and Practices for Perinatal Care Professionals. (DOCX)

S3 Table Content Validation Individual Expert Ratings for Round 2. (DOCX)

S4 Table Face Validity Methodology for the Food Insecurity Questionnaire on Knowledge, Attitudes, and Practices for Perinatal Care Professionals. (DOCX)

S5 Table Face Validation Individual Expert Ratings. (DOCX)

S6 Table Final Version of the Questionnaire on Food Insecurity Knowledge, Attitudes, and Practices for Perinatal Care Professionals, After Face Validation. (DOCX)
