# Supplementary material for: Food insecurity questionnaire on knowledge, attitudes, and practices for perinatal care professionals
Source: PLoS One. 2025 Jul 21;20(7):e0328891. doi: 10.1371/journal.pone.0328891 (PMC12279134; doi:10.1371/journal.pone.0328891)
Supplement: S1 Table — (DOCX) [file pone.0328891.s001.docx]

**Table S1. Initial Version of the Questionnaire on Food Insecurity Knowledge, Attitudes, and Practices for Perinatal Care Professionals, Prior to Round 1 of Content Validation.**

| **Section A. Identification/Screening** | | **Section A. Responses** |
| --- | --- | --- |
| **Q1** | Are you 18 years old or older? | Yes/No |
| **Q2** | Your PLACE OF WORK or CLINIC zip code: 89030, 89031, 89032, 89101, 89106 | 89030, 89031, 89032, 89101, 89106 |
| **Q3** | Do you provide care or services for pregnant people? | Yes/No |
| **Q4** | Do you provide care or services for children under 3 years old? | Yes/No |
| **Q5** | Does the facility where you work accept Medicaid? | Yes/No/I am not a healthcare provider |
| **Section B. Provider Socio-Demographics** | | **Section B. Responses** |
| **S1** | How old are you? | (insert numbers) |
| **S2** | What was the sex assigned to you at birth, on your original birth certificate? | Male, Female, Intersex, Prefer not to answer |
| **S3** | What is your current gender? | Man, Woman, Transgender, Non-binary, Two-Spirit, I use a different term, Prefer not to answer |
| **S4** | What is the highest level of education you have completed? | High school or equivalent (i.e., GED), Technical/vocational/trade school, College degree (including Associate and Bachelor), Graduate degree (Master or Doctorate), Professional degree (e.g., MD, DO, DDS) |
| **S5** | Are you Hispanic or Latino/a/x? | Yes/No |
| **S5** | What race or ethnicity do you consider yourself? | White, Black or African American, Asian, American Indian, Alaskan Native, Native Hawaiian or other Pacific Islander, Biracial/Mixed ethnicity, Other (please specify) |
| **S6** | What language(s) do you speak? | English, Spanish, Other. Which? (.....................) |
| **Section C. Experience and Workplace** | | **Section C. Responses** |
| **W1** | I provide home visits within the eligible zip codes. | Yes (if yes, proceed to question 15)/No (if no, proceed to question 14) |
| **W2** | Within the following zip codes, please indicate the name(s) of the place(s) where you work. | (free text entry) |
| **W3** | What is your employment status at this place? | Full time/Part-time/Casual |
| **W4** | How long have you worked at this place? | Less than 6 months/6 months – 12 months/1-2 years/Over 3 years |
| **W5** | I am a (indicate your primary role) | Pediatrician, OB/GYN, Emergency Medicine, Family Medicine, Internal Medicine, Registered Nurse/Licensed Practical Nurse, Certified Nurse Midwife, Primary care provider/nurse practitioner, Primary care provider/clinical nurse specialist, Primary care provider/physician assistant, Registered Dietitian/Licensed Dietitian Nutritionist , Social Worker, Nutrition Educator, Community Health Worker, Another (specify): |
| **W6** | How long have you been working in your profession? | Less than 1 year/1-2 years/3-5 years/6 years or more |
| **Section D. Provider Knowledge on Food Insecurity**  This section asks questions related to your knowledge of food insecurity and its implications. Knowledge is defined as a set of understandings, knowledge and of “science.” It is also one’s capacity for imagining, one’s way of perceiving. Having knowledge does not automatically mean that one will engage in a certain behavior. There are 17 questions. Please answer honestly. | | **Section D. Knowledge Responses** |
| **K1** | The Hunger Vital Sign™ is a two-question survey to screen for food insecurity. | True/False |
|  |  |  |
| **K2** | Universal screening for food security is not beneficial to all clients/patients. | True/False |
| **K3** | Food assistance programs are available to clients living at risk for food security within this community (e.g., SNAP, WIC, CACFP). | True/False |
|  |  |  |
| **K4** | Food assistance programs are the only available resource to address food insecurity within this community. | True/False |
| **K5** | It is not necessary to refer to WIC when a client/patient is food-insecure and pregnant or caring for a child under 5 years old. | True/False |
| **K6** | Screening positive for food insecurity during pregnancy increases the risk of adverse physical and mental health outcomes (e.g., anxiety, depression, anemia). | True/False |
| **K7** | Screening positive and addressing food insecurity during pregnancy decrease the risk of preterm birth and low infant birth weight. | True/False |
| **K8** | Screening positive and addressing food insecurity during childhood does not impact child development. | True/False |
| **K9** | Screening positive and addressing food insecurity during pregnancy and/or after birth is likely to decrease milk production for breastfeeding (chestfeeding.) | True/False |
| **K10** | Screening for and addressing food insecurity among my clients/patients is likely to positively impact the health and wellbeing of my patients and their families. | True/False |
| **Section D. Provider Attitudes on Food Insecurity**  Attitude is defined as a way of being, a position. These are leanings or “tendencies to….”. This is an intermediate variable between the situation and the response to this situation. There are 9 questions. Please answer honestly. | | **Section D. Attitudes Responses** |
| **A1** | I believe that addressing food insecurity is an important part of my profession. | Disagree, Somewhat disagree, Somewhat agree, Agree |
| **A2** | Providing clients/patients with referrals to food security resources is not in my scope of practice. | Disagree, Somewhat disagree, Somewhat agree, Agree |
| **A3** | I am comfortable screening my clients/patients for food insecurity. | Disagree, Somewhat disagree, Somewhat agree, Agree |
| **A4** | I am comfortable referring my clients/patients to food security resources. | Disagree, Somewhat disagree, Somewhat agree, Agree |
| **A5** | A universal screening tool for food insecurity is/would be useful in my practice. | Disagree, Somewhat disagree, Somewhat agree, Agree |
| **A6** | My workload allows for sufficient time to screen clients/patients for food insecurity. | Disagree, Somewhat disagree, Somewhat agree, Agree |
| **A7** | The electronic medical record system that I use is conducive to universal food insecurity screening. | Disagree, Somewhat disagree, Somewhat agree, Agree |
| **A8** | My clients/patients would trust me more if I screened them for food insecurity. | Disagree, Somewhat disagree, Somewhat agree, Agree |
| **A9** | My clients/patients would trust me more if I provided them with referrals to food security resources. | Disagree, Somewhat disagree, Somewhat agree, Agree |
| **A10** | My clients/patients would trust me more if I followed up with them after referral to food security resources. | Disagree, Somewhat disagree, Somewhat agree, Agree |
| **Section D. Provider Practices on Food Insecurity**  Practices or behaviors are the observable actions of an individual in response to a stimulus. This is something that deals with the concrete, with actions. For practices related to health, one collects information on consumption of tobacco or alcohol, the practice of screening, vaccination practices, sporting activities, sexuality etc. Available responses will be a 5-point Likert scale based on the stages of change: 1 - NO, and I do not intend to in the next 6 months (pre-contemplation), 2 - NO, but I intend to in the next 6 months (contemplation), 3 - NO, but I intend to in the next 30 days (preparation), 4 - YES, I have been, but for LESS than 6 months (action), and 5 - YES, I have been for MORE than 6 months (maintenance). | | **Section D. Practices Responses** |
| **P1** | I screen all pregnant or postpartum clients/patients for food insecurity. | NO, and I do not intend to in the next 6 months; NO, but I intend to in the next 5 months (contemplation); NO, but I intend to in the next 30 days; YES, I have been, but for LESS than 6 months; YES, I have been for MORE than 6 months |
| **P2** | I screen for food insecurity in patients with anemia. | NO, and I do not intend to in the next 6 months; NO, but I intend to in the next 5 months (contemplation); NO, but I intend to in the next 30 days; YES, I have been, but for LESS than 6 months; YES, I have been for MORE than 6 months |
| **P3** | I screen for food insecurity in patients with depression and/or anxiety. | NO, and I do not intend to in the next 6 months; NO, but I intend to in the next 5 months (contemplation); NO, but I intend to in the next 30 days; YES, I have been, but for LESS than 6 months; YES, I have been for MORE than 6 months |
| **P4** | I screen clients/patients (or their caregiver for children up to the age of 3) for food insecurity during all interactions. | NO, and I do not intend to in the next 6 months; NO, but I intend to in the next 5 months (contemplation); NO, but I intend to in the next 30 days; YES, I have been, but for LESS than 6 months; YES, I have been for MORE than 6 months |
| **P5** | I screen for food insecurity when a child age 3 or younger has behavioral problems. | NO, and I do not intend to in the next 6 months; NO, but I intend to in the next 5 months (contemplation); NO, but I intend to in the next 30 days; YES, I have been, but for LESS than 6 months; YES, I have been for MORE than 6 months |
| **P6** | I screen for food insecurity when a patient/client requires a special diet or expensive medication. | NO, and I do not intend to in the next 6 months; NO, but I intend to in the next 5 months (contemplation); NO, but I intend to in the next 30 days; YES, I have been, but for LESS than 6 months; YES, I have been for MORE than 6 months |
| **P7** | I coordinate with community resources to reduce food insecurity among my clients/patients. | NO, and I do not intend to in the next 6 months; NO, but I intend to in the next 5 months (contemplation); NO, but I intend to in the next 30 days; YES, I have been, but for LESS than 6 months; YES, I have been for MORE than 6 months |
| **P8** | I screen for food insecurity during visits for nutrition-related conditions (e.g., diabetes, weight concerns, food allergies). | NO, and I do not intend to in the next 6 months; NO, but I intend to in the next 5 months (contemplation); NO, but I intend to in the next 30 days; YES, I have been, but for LESS than 6 months; YES, I have been for MORE than 6 months |
| **P9** | I refer families experiencing food insecurity to food security services. | NO, and I do not intend to in the next 6 months; NO, but I intend to in the next 5 months (contemplation); NO, but I intend to in the next 30 days; YES, I have been, but for LESS than 6 months; YES, I have been for MORE than 6 months |
| **P10** | After referring a client/patient or family to food security services, I follow up to ensure that food needs are met. | NO, and I do not intend to in the next 6 months; NO, but I intend to in the next 5 months (contemplation); NO, but I intend to in the next 30 days; YES, I have been, but for LESS than 6 months; YES, I have been for MORE than 6 months |
| **P11** | When I feel like I do not have time to screen for food insecurity, I make myself anyway because I know it will make a difference to me and my client/patients. | NO, and I do not intend to in the next 6 months; NO, but I intend to in the next 5 months (contemplation); NO, but I intend to in the next 30 days; YES, I have been, but for LESS than 6 months; YES, I have been for MORE than 6 months |
| **P12** | I schedule meetings and events to educate my clients/patients about food security resources in the community. | NO, and I do not intend to in the next 6 months; NO, but I intend to in the next 5 months (contemplation); NO, but I intend to in the next 30 days; YES, I have been, but for LESS than 6 months; YES, I have been for MORE than 6 months |
| **P13** | I have posters and educational materials about food security visible in areas frequented by clients/patients. | NO, and I do not intend to in the next 6 months; NO, but I intend to in the next 5 months (contemplation); NO, but I intend to in the next 30 days; YES, I have been, but for LESS than 6 months; YES, I have been for MORE than 6 months |
| **P14** | I use a checklist or other form of reminder to prompt me to screen my clients/patients for food insecurity. | NO, and I do not intend to in the next 6 months; NO, but I intend to in the next 5 months (contemplation); NO, but I intend to in the next 30 days; YES, I have been, but for LESS than 6 months; YES, I have been for MORE than 6 months |
