# Supplementary material for: Food insecurity questionnaire on knowledge, attitudes, and practices for perinatal care professionals
Source: PLoS One. 2025 Jul 21;20(7):e0328891. doi: 10.1371/journal.pone.0328891 (PMC12279134; doi:10.1371/journal.pone.0328891)
Supplement: S2 Table — (DOCX) [file pone.0328891.s002.docx]

**Table S2. Content Validity Methodology for the Food Insecurity Questionnaire on Knowledge, Attitudes, and Practices for Perinatal Care Professionals.**

| **Method of Content Validity** | **Definition** | **Acceptable Value** | **Actual Value** |
| --- | --- | --- | --- |
| **Raters** | Target respondents for the finalized questionnaire, general public | 2 to 20 participants or raters | 8 perinatal care professionals working in West and North Las Vegas |
| **Questionnaire Item** |  |  |  |
| **Item Rating** | Raters were asked to rate each item (question) on relevance on a scale of 1 to 4, and given space for additional written feedback (comments) | 3 or 44: Very relevant and succinct 3: Relevant but needs minor alterations  2: Unable to assess relevance without revision  1: Not relevant | **Items rated as 1, Not relevant:** 2**Items rated as 2, Unable to assess without revision:** 0 |
| **Item-Level Content Validity Index**  **(I-CVI)** | Proportion of raters who gave an item a relevance rating of 3 or 4 | Round 1:   - Retained if I-CVI > 0.79 - Needs revision if I-CVI between 0.70 and 0.79 - Deleted if I-CVI < 0.69   Round 2:   - Satisfactory if I-CVI > 0.78 | **Items rated <0.78:** 1 (socio-demographic, item removed) |
| **Item-Level Content Validity Universal Agreement**  **(I-CVI UA)** | Binary score indicating whether all rated scored item as not requiring revision  1 = all raters agree  0 = any rater disagree | 1.0 | **Items rated 0:** 17  **Items rated 1:** 37 |
| **Questionnaire subscales: Knowledge, Attitude, Practices** | | | |
| **Sub-scale-Level Content Validity Index Average**  **(SS-CVI Ave)** | Average of I-CVI scores for each sub-scale (knowledge, attitudes, practices) | 0.80 | **Knowledge:** 0.88  **Attitudes:** 0.96  **Practices:** 0.96 |
| **Sub-scale- Level Content Validity Index Universal Agreement**  **(SS-CVI UA)** | Average of I-CVI equal to 1 for each sub-scale (knowledge, attitudes, practices) | 0.60 | **Knowledge:** 0.41  **Attitudes:** 0.78  **Practices:** 0.80 |
| **Questionnaire full scale** | | | |
| **Scale-Level Content Validity Index Average**  **(S-CVI Ave)** | Average of I-CVI scores for the entire scale | 0.80 | 0.93 |
| **Scale-Level Content Validity Index Universal Agreement**  **(S-CVI UA)** | Average I-CVI equal 1 of all items for the entire scale | 0.60 | 0.70 |

References: Ismail et al., 2020; Gunawan, 2021; Onojakpor & de Kock, 2020; Yusoff, 2019(a); Zamanzadeh et al., 2015
