# Supplementary material for: Food insecurity questionnaire on knowledge, attitudes, and practices for perinatal care professionals
Source: PLoS One. 2025 Jul 21;20(7):e0328891. doi: 10.1371/journal.pone.0328891 (PMC12279134; doi:10.1371/journal.pone.0328891)
Supplement: S3 Table — (DOCX) [file pone.0328891.s003.docx]

**Table S3 Content Validation Individual Expert Ratings for Round 2.**

| **Item** | **Rater 1** | **Rater 2** | **Rater 3** | **Rater 4** | **Rater 5** |  | **Raters in Agreement** | **I-CVI** | **UA** |
| --- | --- | --- | --- | --- | --- | --- | --- | --- | --- |
| **Section 1. Identification and screening** | | | | | | | | | |
| **Q1** | 3 | 4 | 4 | 4 | 4 |  | 4 | 0.8 | 0 |
| **Q2** | 4 | 4 | 4 | 4 | 4 |  | 5 | 1 | 1 |
| **Q3** | 4 | 4 | 4 | 4 | 4 |  | 5 | 1 | 1 |
| **Q4** | 4 | 4 | 4 | 4 | 4 |  | 5 | 1 | 1 |
| **Section 2. Socio-demographic characteristics** | | | | | | | | | |
| **S1** | 4 | 4 | 4 | 4 | 4 |  | 5 | 1 | 1 |
| **S2** | 1 | 4 | 4 | 4 | 3 |  | 3 | 0.6 | 0 |
| **S3** | 4 | 4 | 4 | 4 | 4 |  | 5 | 1 | 1 |
| **S4** | 4 | 4 | 4 | 4 | 4 |  | 5 | 1 | 1 |
| **S5** | 4 | 4 | 4 | 4 | 4 |  | 5 | 1 | 1 |
| **S6** | 4 | 4 | 4 | 4 | 4 |  | 5 | 1 | 1 |
| **S7** | 4 | 4 | 4 | 4 | 4 |  | 5 | 1 | 1 |
| **Section 3. Profession and workplace** | | | | | | | | | |
| **W1** | 4 | 4 | 4 | 4 | 4 |  | 5 | 1 | 1 |
| **W2** | 3 | 4 | 4 | 4 | 4 |  | 4 | 0.8 | 0 |
| **W3** | 4 | 4 | 4 | 4 | 4 |  | 5 | 1 | 1 |
| **W4** | 4 | 4 | 4 | 4 | 4 |  | 5 | 1 | 1 |
| **W5** | 4 | 4 | 4 | 4 | 4 |  | 5 | 1 | 1 |
| **W6** | 3 | 4 | 4 | 4 | 4 |  | 4 | 0.8 | 0 |
| **Section 4. Knowledge** | | | | | | | | | |
| **K1** | 3 | 4 | 4 | 4 | 4 |  | 4 | 0.8 | 0 |
| **K2** | 3 | 4 | 4 | 4 | 4 |  | 4 | 0.8 | 0 |
| **K3** | 4 | 4 | 4 | 4 | 4 |  | 5 | 1 | 1 |
| **K4** | 3 | 4 | 4 | 4 | 4 |  | 4 | 0.8 | 0 |
| **K5** | 3 | 4 | 4 | 4 | 4 |  | 4 | 0.8 | 0 |
| **K6** | 3 | 4 | 4 | 4 | 4 |  | 4 | 0.8 | 0 |
| **K7** | 4 | 4 | 4 | 4 | 4 |  | 5 | 1 | 1 |
| **K8** | 3 | 4 | 4 | 4 | 4 |  | 4 | 0.8 | 0 |
| **K9** | 4 | 4 | 4 | 4 | 4 |  | 5 | 1 | 1 |
| **K10** | 3 | 4 | 4 | 4 | 4 |  | 4 | 0.8 | 0 |
| **K11** | 1 | 4 | 4 | 4 | 4 |  | 4 | 0.8 | 0 |
| **K12** | 3 | 4 | 4 | 4 | 4 |  | 4 | 0.8 | 0 |
| **K13** | 3 | 4 | 4 | 4 | 4 |  | 4 | 0.8 | 0 |
| **K14** | 4 | 4 | 4 | 4 | 4 |  | 5 | 1 | 1 |
| **K15** | 4 | 4 | 4 | 4 | 4 |  | 5 | 1 | 1 |
| **K16** | 4 | 4 | 4 | 4 | 4 |  | 5 | 1 | 1 |
| **K17** | 4 | 4 | 4 | 4 | 4 |  | 5 | 1 | 1 |
| **Section 5. Attitudes** | | | | | | | | | |
| **A1** | 4 | 4 | 4 | 4 | 4 |  | 5 | 1 | 1 |
| **A2** | 3 | 4 | 4 | 4 | 4 |  | 4 | 0.8 | 0 |
| **A3** | 4 | 4 | 4 | 4 | 4 |  | 5 | 1 | 1 |
| **A4** | 3 | 4 | 4 | 4 | 4 |  | 4 | 0.8 | 1 |
| **A5** | 4 | 4 | 4 | 4 | 4 |  | 5 | 1 | 1 |
| **A6** | 4 | 4 | 4 | 4 | 4 |  | 5 | 1 | 1 |
| **A7** | 4 | 4 | 4 | 4 | 4 |  | 5 | 1 | 1 |
| **A8** | 4 | 4 | 4 | 4 | 4 |  | 5 | 1 | 1 |
| **A9** | 4 | 4 | 4 | 4 | 4 |  | 5 | 1 | 1 |
| **A10** | 4 | 4 | 4 | 4 | 4 |  | 5 | 1 | 1 |
| **Section 6. Practices** | | | | | | | | | |
| **P1** | 3 | 4 | 4 | 4 | 4 |  | 4 | 0.8 | 0 |
| **P2** | 3 | 4 | 4 | 4 | 4 |  | 4 | 0.8 | 0 |
| **P3** | 4 | 4 | 4 | 4 | 4 |  | 5 | 1 | 1 |
| **P4** | 4 | 4 | 4 | 4 | 4 |  | 5 | 1 | 1 |
| **P5** | 4 | 4 | 4 | 4 | 4 |  | 5 | 1 | 1 |
| **P6** | 4 | 4 | 4 | 4 | 4 |  | 5 | 1 | 1 |
| **P7** | 4 | 4 | 4 | 4 | 4 |  | 5 | 1 | 1 |
| **P8** | 4 | 4 | 4 | 4 | 4 |  | 5 | 1 | 1 |
| **P9** | 4 | 4 | 4 | 4 | 4 |  | 5 | 1 | 1 |
| **P10** | 4 | 4 | 4 | 4 | 4 |  | 5 | 1 | 1 |
|  | | | | | | | **S-CVI/Ave** | 0.93 |  |
| **Rating as relevant & succinct** | 0.95 | 1 | 1 | 1 | 0.99 |  | **S-CVI/UA** |  | 0.69 |
| **Average proportion of items judged relevant & succinct** | | | | | | 0.99 |  | | |
