# Supplementary material for: Food insecurity questionnaire on knowledge, attitudes, and practices for perinatal care professionals
Source: PLoS One. 2025 Jul 21;20(7):e0328891. doi: 10.1371/journal.pone.0328891 (PMC12279134; doi:10.1371/journal.pone.0328891)
Supplement: S4 Table — (DOCX) [file pone.0328891.s004.docx]

**Table S4 Face Validity Methodology for the Food Insecurity Questionnaire on Knowledge, Attitudes, and Practices for Perinatal Care Professionals.**

| **Method of Face Validity** | **Definition** | **Acceptable Value** | **Actual Value** |
| --- | --- | --- | --- |
| **Raters** | Target population for the finalized questionnaire | 4 to 8+ participants or raters | 8 perinatal care professionals working in West and North Las Vegas |
| **Questionnaire Item** | | | |
| **Item Rating** | Raters were asked to rate each item (question) on clarity, comprehensiveness, and simplicity on a scale of 1 to 4, and given space for additional written feedback (comments) | All three criteria rated as 3 or 4, sum of clarity + comprehensiveness + simplicity scores = 9 to 12 | **Minimum item rated:** 9  **Maximum item rated:** 12 |
| **Item-Level Face Validity Index**  **(I-FVI)** | Proportion of raters who gave an item a clarity, comprehension, and simplicity rating of 3 or 4 | I-FVI = sum of ratings / 12 # of raters | **Minimum:** 0.97  **Maximum:** 1.0 |
| **Item- Level Face Validity Index Universal Agreement**  **(I-FVI UA)** | Binary score indicating whether all raters scored item as not requiring revision  1 = all raters agree  0 = any rater disagrees | 1.0 | **Items rated 1:** 34  **Items rated 0:** 19 |
| **Questionnaire Sub-scale** | | | |
| **Sub-Scale-Level Face Validity Index Average**  **(SS FVI/Ave)** | Average of I-FVI scores for each sub-scale (knowledge, attitudes, practices) | 0.80 | **Knowledge:** 0.99  **Attitudes:** 0.99  **Practices:** 0.97 |
| **Sub-Scale-Level Face Validity Index Universal Agreement**  **(SS FVI/UA)** | Average of I-FVI equal to 1 for each sub-scale (knowledge, attitudes, practices) | 0.60 | **Knowledge:** 0.94  **Attitudes:** 0.88  **Practices:** not available due to the lack of universal agreement |
| **Questionnaire Full Scale** | | | |
| **Scale-Level Face Validity Index Average**  **(S-FVI Ave)** | Average I-FVI scores of all items for the entire scale | 0.80 | 0.99 |
| **Scale-Level Face Validity Index Universal Agreement**  **(S-FVI UA)** | Average I-FVI equal 1 of all items for the entire scale | 0.60 | 0.64 |

References: Cocchi et al., 2023, Gaber & Gaber, 2010; Lau et al., 2017; Moitra et al., 2021; Onojakpor & de Kock, 2020; Yusoff, 2019(b)
