# Supplementary material for: Food insecurity questionnaire on knowledge, attitudes, and practices for perinatal care professionals
Source: PLoS One. 2025 Jul 21;20(7):e0328891. doi: 10.1371/journal.pone.0328891 (PMC12279134; doi:10.1371/journal.pone.0328891)
Supplement: S6 Table — (DOCX) [file pone.0328891.s006.docx]

**Table S6. Final Version of the Questionnaire on Food Insecurity Knowledge, Attitudes, and Practices for Perinatal Care Professionals, After Face Validation.**

| **Section A. Identification/Screening** | | **Section A. Responses** |
| --- | --- | --- |
| **Q1** | Are you 18 years old or older? | Yes/No |
| **Q2** | Is your workplace located within one of these zip codes? 89030, 89031, 89032, 89101, 89106 | Yes - 89030, Yes - 89031, Yes - 89032, Yes - 89101, Yes- 89106, I travel to provide services in at least one of these zip codes, I am adjacent to and provide services for clients/patients from at least one of these zip codes, No |
| **Q3** | Do you directly provide care or services for pregnant people and/or children under 3 years old (e.g., medical care, education, day care)? | Yes/No |
| **Q4** | What is your primary workplace role? | Community health worker, Doula, Early childhood educator, Lactation consultant, Nutrition educator, OB/GYN, Parenting educator, Pediatrician, Primary care provider, Certified midwife (such as certified nurse-midwife or certified professional midwife), Traditional midwife, Registered Nurse/Licensed Practical Nurse, Registered Dietitian/Licensed Dietitian Nutritionist, Other (please specify): _________ |
| **Section B. Provider Socio-Demographics** | | **Section B. Responses** |
| **S1** | How old are you? | (insert numbers) |
| **S2** | What is your current gender? | Man, Woman, Transgender, Non-binary, Two-Spirit, I use a different term, Prefer not to answer |
| **S3** | What is the highest level of education you have completed? | Less than high school, High school or equivalent (i.e., GED), Technical/vocational/trade school, College degree (including Associate and Bachelor), Graduate degree (Master or Doctorate), Professional degree (e.g., MD, DO, DDS) |
| **S4** | Are you of Hispanic, Latino, or Spanish origin? | Yes/No/Prefer not to answer |
| **S5** | What is your race? Check all that apply. | White, Black or African American, Asian, American Indian or Alaska Native, Native Hawaiian or other Pacific Islander, Prefer not to answer, Some other race (please specify): _________ |
| **S6** | Do you speak a language other than English? | No/Yes (please specify): _______ |
| **Section C. Experience and Workplace** | | **Section C. Responses** |
| **W1** | Do you provide home visits (i.e., a midwife who travels to and provides services in a personal residence)? | Yes/No |
| **W2** | What is the name of your primary workplace? | Nevada Health Center (89030), Nevada Health Center (89106), Nevada Silver State Stars, Paiute Tribe HHS, SNHD: maternal-child health (such as Embracing Healthy Baby, Nurse-Family Partnership, Thrive 0-3), Sunrise Children's Hospital, WIC: Catholic Charities, WIC: MLK, WIC: North Las Vegas, Other (please specify): __________ |
| **W3** | What is your employment status at this place? | Full-time, Part-Time, Casual (contract, as-needed basis), Volunteer |
| **W4** | How long have you worked at this place? | Less than 6 months/6 months - 11 months/1-2 years/3 years or more |
| **W5** | How long have you been working in this profession? | Less than 1 year/1-2 years/3-5 years/6 years or more |
| **Section D. Provider Knowledge on Food Insecurity**  This section asks questions related to your knowledge of food insecurity and its implications. Knowledge is defined as a set of understandings, knowledge and of “science.” It is also one’s capacity for imagining, one’s way of perceiving. Having knowledge does not automatically mean that one will engage in a certain behavior. There are 17 questions. Please answer honestly. | | **Section D. Knowledge Responses** |
| **K1** | It is possible to screen households for food insecurity. | True/False |
| **K2** | I am aware of the Hunger Vital Sign™, a validated food insecurity screening tool that consists of two questions. | True/False |
| **K3** | Screening for food insecurity may not be beneficial to all my clients/patients. | True/False |
| **K4** | Offices to enroll in food assistance programs (e.g., WIC) are available to my clients/patients within this community. | True/False |
| **K5** | For clients/patients already enrolled in food assistance programs (e.g., WIC), they are able to access food within the community thanks to these resources. | True/False |
| **K6** | Organizations providing emergency food resources (e.g., food banks) are available within this community. | True/False |
| **K7** | When a client/patient is food-insecure and pregnant or caring for a child under 5 years, I should refer them to enrollment in food assistance programs (e.g., WIC). | True/False |
| **K8** | Experiencing food insecurity during pregnancy increases the risk of adverse physical and mental health outcomes (e.g. anemia, anxiety, depression). | True/False |
| **K9** | Addressing food insecurity during pregnancy may decrease the risk of preterm birth and low infant birth weight. | True/False |
| **K10** | Lactation support provided by food assistance programs (i.e., WIC) may reduce food insecurity among infants. | True/False |
| **K11** | Formula feeding instead of breast/chestfeeding among low-income families may increase risk of infant food insecurity, especially during formula shortages. | True/False |
| **K12** | Experiencing food insecurity during pregnancy and/or after birth is likely to decrease milk production for breast/chestfeeding. | True/False |
| **K13** | Addressing food insecurity during childhood will not have a positive impact on child development. | True/False |
| **K14** | It is not important to screen for food insecurity during patient/client visits for nutrition-related conditions (e.g., diabetes, weight concerns, food allergies). | True/False |
| **K15** | It is not important to screen for food insecurity when a child age 3 or younger has behavioral problems. | True/False |
| **K15** | It is not important to screen for food insecurity when a patient/client requires a special diet. | True/False |
| **K17** | It is not important to screen for food insecurity when a patient/client requires expensive medication. | True/False |
| **Section D. Provider Attitudes on Food Insecurity**  Attitude is defined as a way of being, a position. These are leanings or “tendencies to….”. This is an intermediate variable between the situation and the response to this situation. There are 9 questions. Please answer honestly. | | **Section D. Attitudes Responses** |
| **A1** | Screening for and addressing food insecurity is likely to positively impact the physical and mental health of my clients/patients and their families. Therefore, I am ready to do my part. | Disagree, Somewhat disagree, Somewhat agree, Agree |
| **A2** | Providing clients/patients with referrals to food assistance programs (e.g., WIC) and/or emergency food resources (e.g., food banks) is not in my scope of practice. | Disagree, Somewhat disagree, Somewhat agree, Agree |
| **A3** | I am comfortable screening my clients/patients for food insecurity. | Disagree, Somewhat disagree, Somewhat agree, Agree |
| **A4** | I am comfortable referring my clients/patients to food assistance programs (e.g., WIC) and/or emergency food resources (e.g., food banks). | Disagree, Somewhat disagree, Somewhat agree, Agree |
| **A5** | A universal screening tool for food insecurity is/would be useful in my practice. | Disagree, Somewhat disagree, Somewhat agree, Agree |
| **A6** | Universal screening for food insecurity interferes/would interfere with my routine. | Disagree, Somewhat disagree, Somewhat agree, Agree |
| **A7** | My clients/patients would trust me more if I screened them for food insecurity. | Disagree, Somewhat disagree, Somewhat agree, Agree |
| **A8** | My clients/patients would trust me more if I provided them with referrals to community resources to reduce food insecurity. | Disagree, Somewhat disagree, Somewhat agree, Agree |
| **A9** | My clients/patients would trust me more if I followed up with them after referral to community resources to reduce food insecurity. | Disagree, Somewhat disagree, Somewhat agree, Agree |
| **Section D. Provider Practices on Food Insecurity**  Practices or behaviors are the observable actions of an individual in response to a stimulus. This is something that deals with the concrete, with actions. For practices related to health, one collects information on consumption of tobacco or alcohol, the practice of screening, vaccination practices, sporting activities, sexuality etc. Available responses will be a 5-point Likert scale based on the stages of change: 1 - NO, and I do not intend to in the next 6 months (pre-contemplation), 2 - NO, but I intend to in the next 6 months (contemplation), 3 - NO, but I intend to in the next 30 days (preparation), 4 - YES, I have been, but for LESS than 6 months (action), and 5 - YES, I have been for MORE than 6 months (maintenance). | | **Section D. Practices Responses** |
| **P1** | I screen all pregnant or postpartum clients/patients for food insecurity. | NO, and I do not intend to in the next 6 months; NO, but I intend to in the next 6 months; NO, but I intend to in the next 30 days; YES, I have been, but for LESS than 6 months; YES, I have been for MORE than 6 months |
| **P2** | I screen clients/patients or their caregiver for children up to the age of 3 years for food insecurity during all interactions. | NO, and I do not intend to in the next 6 months; NO, but I intend to in the next 6 months; NO, but I intend to in the next 30 days; YES, I have been, but for LESS than 6 months; YES, I have been for MORE than 6 months |
| **P3** | I use an electronic record system to screen for food insecurity. | NO, and I do not intend to in the next 6 months; NO, but I intend to in the next 6 months; NO, but I intend to in the next 30 days; YES, I have been, but for LESS than 6 months; YES, I have been for MORE than 6 months |
| **P4** | I coordinate with community resources to reduce food insecurity among my clients/patients. | NO, and I do not intend to in the next 6 months; NO, but I intend to in the next 6 months; NO, but I intend to in the next 30 days; YES, I have been, but for LESS than 6 months; YES, I have been for MORE than 6 months |
| **P5** | I refer families experiencing food insecurity to food assistance programs (e.g., WIC) and/or emergency food resources (e.g., food banks). | NO, and I do not intend to in the next 6 months; NO, but I intend to in the next 6 months; NO, but I intend to in the next 30 days; YES, I have been, but for LESS than 6 months; YES, I have been for MORE than 6 months |
| **P6** | After referring a client/patient or family to food assistance programs (e.g., WIC) and/or emergency food resources (e.g., food banks), I follow up to ensure that food needs are met. | NO, and I do not intend to in the next 6 months; NO, but I intend to in the next 6 months; NO, but I intend to in the next 30 days; YES, I have been, but for LESS than 6 months; YES, I have been for MORE than 6 months |
| **P7** | When I feel like I do not have time to screen for food insecurity, I make myself anyway because I know it will make a difference to my clients/patients. | NO, and I do not intend to in the next 6 months; NO, but I intend to in the next 6 months; NO, but I intend to in the next 30 days; YES, I have been, but for LESS than 6 months; YES, I have been for MORE than 6 months |
| **P8** | I schedule meetings and events to educate my clients/patients in the community about food assistance programs (e.g., WIC) and/or emergency food resources (e.g., food banks) to reduce food insecurity. | NO, and I do not intend to in the next 6 months; NO, but I intend to in the next 6 months; NO, but I intend to in the next 30 days; YES, I have been, but for LESS than 6 months; YES, I have been for MORE than 6 months |
| **P9** | I have posters and educational materials about food insecurity visible in areas frequented by clients/patients. | NO, and I do not intend to in the next 6 months; NO, but I intend to in the next 6 months; NO, but I intend to in the next 30 days; YES, I have been, but for LESS than 6 months; YES, I have been for MORE than 6 months |
| **P10** | I use a checklist or other form of reminder to prompt me to screen my clients/patients for food insecurity. | NO, and I do not intend to in the next 6 months; NO, but I intend to in the next 6 months; NO, but I intend to in the next 30 days; YES, I have been, but for LESS than 6 months; YES, I have been for MORE than 6 months |
